# When can we compute analytically lookback time, age of the universe, and luminosity distance?

**DOI:** 10.1140/epjc/s10052-022-10519-2

**Published:** 2022-06-24

**Authors:** Sonia Jose, Alexandre Leblanc, Valerio Faraoni

**Affiliations:** 1 Department of Physics and Astronomy, Bishop’s University, 2600 College Street, Sherbrooke, QC J1M 1Z7 Canada; 2 Department of Physics, Université de Sherbrooke, 2500 Boulevard de l’Université, Sherbrooke, QC J1K 2R1 Canada

## Abstract

In Friedmann–Lemaître–Robertson–Walker cosmology, it is sometimes possible to compute analytically lookback time, age of the universe, and luminosity distance versus redshift, expressing them in terms of a finite number of elementary functions. We classify these situations using the Chebyshev theorem of integration and providing examples.

## Introduction

When can we compute *exactly* lookback time, age of the universe, and redshift-luminosity distance relation $D_L(z)$ in Friedmann–Lemaître–Robertson–Walker (FLRW)  cosmology? The age of the universe $t_0$ sets an upper bound on the present value $H_0$ of the Hubble function, with implications for the current Hubble tension [1]. Lookback time, age of the universe, and redshift-luminosity distance relation, of crucial importance for modern cosmology, are expressed by integrals taking the form of infinite hypergeometric series. Of course, lookback time, age, and luminosity distance can always be computed numerically in a given cosmological model, however one would also like to know when they can be computed analytically in terms of a finite number of elementary functions. This simplification happens when the hypergeometric series expressing them truncate. Equivalently, it happens when the integral expressing lookback time, age, or luminosity distance is of a special form contemplated by the Chebyshev theorem of integration [2, 3]. The truncation of the hypergeometric series, or the equivalent Chebyshev theorem, were used in the 1960s [4–7], and were recently rediscovered [8], to derive two- and three-fluid (or effective fluid) analytical solutions of the Einstein–Friedmann equations (see [9] for a review).

The objective of the present paper is not to propose an alternative method to compute the age of the universe or the luminosity distance in the situations relevant for the observed universe in which these expressions are already known (the computation method remains the same in these cases). The goal is rather to identify and classify the situations, which for various reasons may be of interest to theorists, in which the corresponding integrals can be expressed exactly in simple form.

When the matter content of the FLRW universe consists of at most three non-interacting fluids or effective fluids (which includes spatial curvature and/or the cosmological constant $\Lambda$, if present), and assuming that the equations of state of these fluids are of the form $P=w\rho$ with *w* constant and rational, the situations in which lookback time, age, and luminosity distance are analytical and simple are classified by means of the Chebyshev theorem [2, 3].

The assumption that the equation of state parameter *w* be a rational number is not restrictive. First, this is almost always a rational number in the cosmological literature [10–13]. Second, even if *w* is irrational, in practice cosmological observations cannot distinguish between *w* and its rational approximation and it is an excellent approximation to replace the actual value of this parameter with a rational approximation to it containing a sufficient number of digits.

Sections 2 and 3 catalogue situations in which the universe is characterized by two or three fluids or effective fluids (which includes cosmological constant and spatial curvature) and lookback time and age are computed analytically in simple form. Section 4 reports situations in which one can compute analytically the luminosity distance $D_\mathrm {L}(z)$ as a function of redshift *z*. Section 5 contains concluding remarks.

We follow the notation and conventions of Ref. [10]: the metric signature is ${-}{+}{+}{+}$, *G* is Newton’s constant, and units are used in which the speed of light *c* is unity.

## Lookback time and age of the universe

We consider a homogeneous and isotropic universe described by the FLRW line element in spherical comoving coordinates $\left( t,r, \vartheta ,\varphi \right)$

1 $$\begin{aligned} ds^2=-dt^2 +a^2(t)\left( \frac{dr^2}{1-Kr^2} + r^2 d\Omega _{(2)}^2 \right) \end{aligned}$$

where $d\Omega _{(2)}^2=d\vartheta ^2 +\sin ^2 \vartheta \, d\varphi ^2$ is the line element on the unit 2-sphere, *K* is the curvature index (which we take to be normalized to $0, \pm 1$), and *a*(*t*) is the scale factor. We assume that the matter source of the FLRW universe is a perfect fluid with energy density $\rho$ and pressure *P* related by the barotropic, linear, and constant equation of state

2 $$\begin{aligned} P=w\rho , \quad \quad w=\text{ const. } \end{aligned}$$

The Einstein–Friedmann equations describing the evolution of this universe read

3 $$\begin{aligned}&H^2= \frac{8\pi G}{3} \, \rho +\frac{\Lambda }{3}-\frac{K}{a^2}, \end{aligned}$$

4 $$\begin{aligned}&\frac{\ddot{a}}{a} = -\frac{4\pi G}{3} \left( \rho +3P \right) +\frac{\Lambda }{3}, \end{aligned}$$

5 $$\begin{aligned}&{\dot{\rho }}+3H\left( P+\rho \right) =0, \end{aligned}$$

where an overdot denotes differentiation with respect to the comoving time *t*, $H \equiv {\dot{a}}/a$ is the Hubble function, and $\Lambda$ is the cosmological constant. The conservation equation (5) gives

6 $$\begin{aligned} \rho (a)= \rho _0 \left( \frac{a_0}{a} \right) ^{3(w+1)}, \end{aligned}$$

where $$\rho _{0}$$ is a constant. Suppose that the cosmic fluid is a mixture of *n* non-interacting fluids with individual densities $$\rho _{i}$$ and pressures $$P_i$$, with $$ P_i = w_i \rho _i $$ and $$ w_i=$$ const. ($$i=1,2,3, \ldots , n$$). The total energy density and pressure are

7 $$\begin{aligned} \rho _\mathrm {(tot)}=\sum _{i=1}^n \rho _i, \quad \quad P_\mathrm {(tot)}= \sum _{i=1}^n w_i \rho _i, \end{aligned}$$

respectively.

We consider universes beginning with a Big Bang $a(0)=0$ at $t=0$ and we denote the present value of time-dependent quantities with a zero subscript. Then, since ${\dot{a}} = da/dt$, the lookback time to a source that emitted at time $t_e$, scale factor $a_e$, and redshift $z_e$ is

8 $$\begin{aligned} t_L = \int _{t_e}^t dt'= \int _{a_e}^{a_0} \frac{da'}{ {\dot{a}}' } \end{aligned}$$

with ${\dot{a}}$ given by the Friedmann equation (3). In the limit $t_e \rightarrow 0$ (or $a_e\rightarrow 0$, or $z_e\rightarrow +\infty$), one obtains the age of the universe

9 $$\begin{aligned} t_0 = \int _0^t dt'= \int _0^{a_0} \frac{da'}{ {\dot{a}}' }. \end{aligned}$$

Rewrite the Friedmann equation as

10 $$\begin{aligned} \frac{K}{a^2} =H^2 \left( \Omega ^\mathrm {(tot)} +\Omega _{\Lambda } -1 \right) , \end{aligned}$$

where $\Omega ^\mathrm {(tot)}$ is the total energy density of the real fluids (as opposed to the *effective* fluids given by $\Lambda$ and by the curvature term) in units of the critical density $\rho _c \equiv \frac{3H^2}{8\pi G}$. For a single fluid, using Eq. (6) one obtains

11 $$\begin{aligned} {\dot{a}}&= \sqrt{\frac{8\pi G}{3} \, a_0^2 \rho _0 \left( \frac{a_0}{a}\right) ^{3w+1} - \left( \Omega _0 -1\right) a_0^2 H_0^2 +\frac{\Lambda a_0^2}{3} \left( \frac{a_0}{a}\right) ^{-2} } \nonumber \\&= a_0 H_0 \sqrt{ 1-\Omega _0^\mathrm {(tot)} + \Omega _0 \left( z+1 \right) ^{3w+1} + \Omega _{\Lambda 0} \left( z+1 \right) ^{-2} }, \end{aligned}$$

where $z \equiv a_0/a -1$ is the redshift factor, and then

12 $$\begin{aligned} t_L= & {} H_0^{-1} \int _0^{z_e} dz' \left( z'+1\right) ^{-2} \left[ 1-\Omega _0^\mathrm {(tot)}+\Omega _0 \left( z'+1\right) ^{3w+1} \right. \nonumber \\&\left. +\Omega _{\Lambda 0}\left( z'+1\right) ^{-2} \right] ^{-1/2}. \end{aligned}$$

The change of variable $z \rightarrow x \equiv a/a_0 = \left( 1+z \right) ^{-1}$ in the integral turns it into

13 $$\begin{aligned} t_L =H_0^{-1} \int _{x_e}^1 \frac{dx}{\sqrt{1-\Omega _0^\mathrm {(tot)} + \Omega _0 \, x^{ -(3w+1)} + \Omega _{\Lambda 0} \, x^2 } }. \end{aligned}$$

For suitable values of the equation of state parameter *w*, this integral can be expressed in terms of elementary functions using the Chebyshev theorem of integration [2, 3]:

*The integral*

14 $$\begin{aligned} J \equiv \int dx \, x^p \Big ( \alpha + \beta x^r\Big )^q, \quad \quad \quad r\ne 0, \quad p,q,r \in {\mathbb {Q}} \end{aligned}$$

*admits a representation in terms of elementary functions if and only if at least one of*  $\frac{p+1}{r}$,  *q*,  $\frac{p+1}{r}+q$ *is an integer.*

In order for the integral in Eq. (13) to be of the Chebyshev form, one of the following possibilities needs to be realized.

### $K = 0$, $\Lambda \ne 0$, plus a single fluid

Suppose that the universe is sourced by a single fluid with equation of state parameter *w* and has non-zero cosmological constant $\Lambda$. This situation includes the $\Lambda$-Cold Dark Matter ($\Lambda$CDM) model if the fluid is a dust. Spatial flatness $K=0$ is equivalent to $\Omega _0^\mathrm {(tot)} =1$ and the integral in Eq. (13) has the form^1^

15 $$\begin{aligned} t_L \, H_0 = \int _{x_e}^1 dx \, x^{-1} \left[ \Omega _{0} \, x^{-3(1+w)} +\Omega _{\Lambda 0} \right] ^{-1/2} \end{aligned}$$

i.e, the form (14) with

16 $$\begin{aligned} p=-1, \quad \quad r=-3(1+w)\ne 0, \quad \quad q=-1/2, \end{aligned}$$

which all are rational if *w* is. Most values of *w* considered in the cosmological literature are rational but, in any case, cosmological observations cannot distinguish between an irrational value of *w* and its rational approximation, hence in practice one can always assume $w\in {\mathbb {Q}}$. The Chebyshev theorem applies since $\frac{p+1}{r} = 0$. Indeed, a direct computation of the integral gives the lookback time (see  Appendix A)17In the limit $x_e\rightarrow 0$, $t_L\rightarrow 0$ and we obtain the age of the universe (see  Appendix A)

18 $$\begin{aligned} t_0 =\frac{2H_0^{-1}}{3(w+1) \sqrt{\Omega _{\Lambda 0}}} \, \ln \Bigg ( \frac{ 1+ \sqrt{\Omega _{\Lambda 0}} }{\sqrt{ 1- \Omega _{\Lambda 0}} } \Bigg ). \end{aligned}$$

This formula appears in textbooks (e.g., [11, 12]) for the special case $w=0$ of dust but it does not seem to be known for general values of *w*.

### $\Lambda =0$, single fluid plus spatial curvature

This situation also leads to physically interesting scenarios. Some of these universes contain dust or radiation and are found in cosmology textbooks.

For a single fluid with equation of state parameter *w* and spatial curvature ($K = \pm 1$), the lookback time (13) is

19 $$\begin{aligned} t_0 = H_0^{-1} \int _{x_e}^1 dx \, \left[ 1-\Omega _0^\mathrm {(tot)} + \Omega _0 \, x^{-\left( 3w+1 \right) } \right] ^{-1/2}. \end{aligned}$$

Comparing with Eq.  (14) yields the exponents

20 $$\begin{aligned} p=0, \quad \quad r=-(3w+1), \quad \quad q=-1/2, \end{aligned}$$

which are all rational if $w \in {\mathbb {Q}}$. The conditions for the Chebyshev theorem to hold are

21 $$\begin{aligned} \frac{p+1}{r}=\frac{-1}{3w+1} = n \end{aligned}$$

or

22 $$\begin{aligned} \frac{p+1}{r}+q=\frac{-3(w+1)}{2(3w +1)} = m, \end{aligned}$$

where $n, m \in {\mathbb {Z}}$. The possible values of *w* for this to happen are the countable infinities of values

23 $$\begin{aligned} w_n =- \frac{(n+1)}{3n} \end{aligned}$$

and

24 $$\begin{aligned} w_m =-\frac{(3+2m)}{3(1+2m)}. \end{aligned}$$

Only a few of the equation of state parameters thus obtained are of physical interest. Focusing on the first possibility (23), as *n* spans the values $n=-\infty , \ldots , -3, -2, -1, 1, 2, 3, \ldots , +\infty$, one obtains

25 $$\begin{aligned} w_n =-\frac{1}{3}, \ldots , -\frac{2}{9}, -\frac{1}{6},\;\;0, - \frac{ 2}{3}, -\frac{1}{2}, - \frac{4}{9}, \ldots , -\frac{1}{3}. \end{aligned}$$

By imposing the second condition (24), as *m* spans the range $-\infty , \ldots , -3, -2, -1, 1, 2, 3, \ldots , +\infty$, one obtains

26 $$\begin{aligned} w_m=-\frac{1}{3}, \ldots , -\frac{1}{5}, -\frac{1}{9},\;\;\frac{1}{3},\;\; -1, - \frac{5}{9}, -\frac{7}{15}, \ldots , -\frac{1}{3}. \end{aligned}$$

The values $w=0$ (dust), $w=1/3$ (radiation), and $w=-1/3$ (empty space with a hyperbolic foliation, i.e., the Milne universe) correspond to textbook cases [10–13]. For dust, they give the well-known Mattig relation^2^ [15]

27 $$\begin{aligned} D_L(z)= & {} \frac{2H_0^{-1} }{\Omega _0^\mathrm {(dust)} }\Bigg [ \Omega _0^\mathrm {(dust)} \, z\left( 2- \Omega _0^\mathrm {(dust)} \right) \nonumber \\&- \left( \sqrt{1+\Omega _0^\mathrm {(dust)}\, z} -1 \right) \Bigg ]. \end{aligned}$$

The values of *w* different from $0, \pm 1/3$ found above describe phantom or quintessence fluids that, although unrealistic to describe the present universe, could be used as toy models for theoretical purposes. The degenerate case $w =-1$ reproduces the empty universe with cosmological constant and spatial curvature.

## Lookback time and age of a spatially flat universe with two (real or effective) fluids

Consider the case of two fluids with equations of state $P_1=w_1\rho _1$ and $P_2=w_2\rho _2$, with $w_{1,2} =$ constants. It is assumed that these two fluids have the same four-velocity $u^{c}$ in their stress-energy tensors. We regard cosmological constant and spatial curvature term as effective fluids hence, in the following, certain conditions correspond to the possibility of one or both fluids being the curvature- or the $\Lambda$- (effective) fluids. The total fluid density is $\rho _\mathrm {(tot)}=\rho _{1}+\rho _{2}$ and the individual densities scale as

28 $$\begin{aligned} \rho _1= \rho _1^{(0)} \left( \frac{a_0}{a} \right) ^{3(w_1+1)}, \quad \quad \rho _2= \rho _2^{(0)} \left( \frac{a_0}{a} \right) ^{3(w_2+1)}, \end{aligned}$$

then the integral in Eq. (13) is

29 $$\begin{aligned} t_0 H_0 = \int _0^1 dx \, x^{(3w_1+1)/2} \left[ \Omega _{0}^{(1)} + \Omega _{0}^{(2)} \, x^{3(w_1 - w_2)} \right] ^{-1/2} \end{aligned}$$

and comparison with Eq.  (14) yields the exponents

30 $$\begin{aligned} p=\frac{3w_1 + 1}{2}, \quad \quad r=3(w_1 - w_2), \quad \quad q=-1/2, \end{aligned}$$

with $w_1\ne w_2$. The conditions for the Chebyshev theorem to hold are

31 $$\begin{aligned} \frac{p+1}{r}=\frac{w_1+1}{2(w_1-w_2)} = n \in {\mathbb {Z}} \end{aligned}$$

or

32 $$\begin{aligned} \frac{p+1}{r}+q=\frac{w_2+1}{2(w_1-w_2)} = m \in {\mathbb {Z}}. \end{aligned}$$

### First condition: $\frac{p+1}{r}=n \in {\mathbb {Z}}$

The first integrability condition (31) gives

33 $$\begin{aligned} \left( 2n-1\right) w_1 -2nw_2 =1 \quad \quad \text{ if } \,\, w_1\ne w_2 \end{aligned}$$

(the case $w_1=w_2$ corresponds to $n=\pm \infty$). Fixing the first fluid (i.e., $w_1\in {\mathbb {Q}}$) yields integrable cases by varying *n*.

#### Dust plus a second (real or effective) fluid

If the first fluid is a dust with $w_1=0$, “simple” integrability cases are obtained when

34 $$\begin{aligned} w_2=-\frac{1}{2n}; \end{aligned}$$

as $n=-\infty , \ldots , -3, -2, -1, 1, 2, 3, \ldots , +\infty$, we obtain the pairs

35 $$\begin{aligned} \begin{array}{rcll} \left( w_1, w_2 \right) &{}=&{} \left( 0, 0 \right) &{} \text{(single } \text{ dust } \text{ fluid) },\\ &{}&{}&{}\\ &{} \ldots &{} &{} \\ &{}&{}&{}\\ \left( w_1, w_2 \right) &{}=&{} \left( 0, \pm \frac{1}{6} \right) ,&{} \\ &{}&{}&{}\\ \left( w_1, w_2 \right) &{}=&{} \left( 0, \pm \frac{1}{4} \right) ,&{}\\ &{}&{}&{}\\ \left( w_1, w_2 \right) &{}=&{} \left( 0, \pm \frac{1}{2} \right) ,&{}\\ &{}&{}&{}\\ &{} \ldots &{} &{}\\ &{}&{}&{}\\ \left( w_1, w_2 \right) &{}=&{} \left( 0 , 0\right) &{} \text{(again, } \text{ a } \text{ single } \text{ dust } \text{ fluid) }. \end{array} \end{aligned}$$

Most of the analytical cases shown above do not have much physical relevance. Although quintessence models are present, the values of $w_2$ are not close to the value $-1$ measured by current observations. The limit $n\rightarrow \pm \infty$ produces a second dust, i.e., there is a single dust fluid in the FLRW universe and integration is trivial.

#### Radiation plus a second (real or effective) fluid

If the first fluid is radiation, $w_1=1/3$, the corresponding values of $w_2$ for integrability à la Chebyshev are

36 $$\begin{aligned} w_2=\frac{n-2}{3n}, \end{aligned}$$

producing the pairs

37 $$\begin{aligned} \begin{array}{rcll} \left( w_1, w_2\right) &{}=&{} \left( \frac{1}{3} , \frac{1}{3} \right) &{}\text{(a } \text{ single } \text{ radiation } \text{ fluid) }, \\ &{}&{}&{}\\ &{} \ldots &{} &{}\\ &{}&{}&{}\\ \left( w_1, w_2 \right) &{}=&{} \left( \frac{1}{3} , \frac{5}{9} \right) ,&{}\\ &{}&{}&{}\\ \left( w_1, w_2 \right) &{}=&{} \left( \frac{1}{3} , \frac{2}{3} \right) ,&{}\\ &{}&{}&{}\\ \left( w_1, w_2 \right) &{}=&{} \left( \frac{1}{3} , 1 \right) &{}\text{(radiation } \text{ plus } \text{ stiff } \text{ fluid) }, \\ &{}&{}&{}\\ \left( w_1, w_2 \right) &{}=&{} \left( \frac{1}{3} , -\frac{1}{3} \right) &{} \text{(radiation } \text{ plus } \text{ spatial } \text{ curvature) },\\ &{}&{}&{}\\ \left( w_1, w_2 \right) &{}=&{} \left( \frac{1}{3} , 0 \right) &{} \text{(radiation } \text{ plus } \text{ dust) },\\ &{}&{}&{}\\ \left( w_1, w_2 \right) &{}=&{} \left( \frac{1}{3} , \frac{1}{9} \right) ,&{}\\ &{}&{}&{}\\ &{} \ldots &{} &{}\\ &{}&{}&{}\\ \left( w_1, w_2 \right) &{}=&{} \left( \frac{1}{3} , \frac{1}{3} \right) &{}\text{(again, } \text{ a } \text{ single } \text{ radiation } \text{ fluid) }. \end{array} \end{aligned}$$

#### Cosmological constant plus a second (real or effective) fluid

The value $w_1=-1$ corresponds to $n=0$ in Eq. (31) and is satisfied by any value of $w_2 \ne -1$, producing a cosmological constant with any single perfect fluid.

#### Stiff matter plus a second (real or effective) fluid

In this case $w_1=1$,

38 $$\begin{aligned} w_2= \frac{n-1}{n}, \end{aligned}$$

and we have the pairs

39 $$\begin{aligned} \begin{array}{rcll} \left( w_1, w_2 \right) &{}=&{} \left( 1, 1 \right) &{} \;\;\;\;\text{(single } \text{ stiff } \text{ fluid) },\\ &{}&{}&{}\\ &{} \ldots &{} &{}\\ &{}&{}&{}\\ \left( w_1, w_2 \right) &{}=&{} \left( 1, \frac{4}{3} \right) ,&{} \\ &{}&{}&{}\\ \left( w_1, w_2 \right) &{}=&{} \left( 1, \frac{3}{2} \right) ,&{}\\ &{}&{}&{}\\ \left( w_1, w_2 \right) &{}=&{} \left( 1, 2\right) ,&{}\\ &{}&{}&{}\\ \left( w_1, w_2 \right) &{}=&{} \left( 1, 0 \right) &{} \text{(stiff } \text{ matter } \text{ plus } \text{ dust) },\\ &{}&{}&{}\\ \left( w_1, w_2 \right) &{}=&{} \left( 1, \frac{1}{2} \right) ,&{}\\ &{}&{}&{}\\ \left( w_1, w_2 \right) &{}=&{} \left( 1, \frac{2}{3} \right) ,&{}\\ &{}&{}&{}\\ &{} \ldots &{} \\ &{}&{}&{}\\ \left( w_1, w_2 \right) &{}=&{} \left( 1 , 1 \right) &{}\text{(again, } \text{ a } \text{ single } \text{ stiff } \text{ fluid) }. \end{array} \end{aligned}$$

The physically most relevant situation is that of a stiff fluid plus dust.

### Second condition: $\frac{p+1}{r} +q =m \in {\mathbb {Z}}$

The second condition (32) yields

40 $$\begin{aligned} w_2=\frac{2m w_1 -1}{2m+1} \quad \text{ if } \, w_2 \ne w_1; \end{aligned}$$

as done for the first condition, we fix the first fluid (i.e., $w_1\in {\mathbb {Q}}$), and we obtain integrable cases as *m* varies.

#### Dust plus a second (real or effective) fluid

If the first fluid is a dust, $w_1=0$, we have

41 $$\begin{aligned} w_2=-\frac{1}{1+2m} \end{aligned}$$

and the pairs

42 $$\begin{aligned} \begin{array}{rcll} \left( w_1, w_2\right) &{}=&{} \left( 0 ,0 \right) &{} \text{(a } \text{ single } \text{ dust } \text{ fluid) },\\ &{} \ldots &{} &{} \\ \left( w_1, w_2\right) &{}=&{} \left( 0 , \pm \frac{1}{5} \right) ,\\ \left( w_1, w_2\right) &{}=&{} \left( 0 , \pm \frac{1}{3} \right) &{} \text{(dust } \text{ plus } \text{ radiation } \text{ or } \text{ curvature) }, \\ \left( w_1, w_2\right) &{}=&{} \left( 0 , \pm 1 \right) &{} (\text{ dust } \text{ plus } \text{ stiff } \text{ fluid } \text{ or } \; \Lambda ),\\ &{} \ldots &{} &{}\\ \left( w_1, w_2\right) &{}=&{} \left( 0 , 0 \right) &{} \text{(again, } \text{ a } \text{ single } \text{ dust } \text{ fluid) }. \end{array} \nonumber \\ \end{aligned}$$

Physically plausible combinations include dust and stiff fluid, dust and radiation, dust and spatial curvature.

#### Radiation plus a second (real or effective) fluid

Beginning with radiation $w_1=1/3$, we obtain

43 $$\begin{aligned} w_2=\frac{2m-3}{3(1+2m)} \end{aligned}$$

and the pairs

44 $$\begin{aligned} \begin{array}{rcll} \left( w_1, w_2\right) &{}=&{} \left( \frac{1}{3} , \frac{1}{3} \right) &{} \text{(single } \text{ radiation } \text{ fluid } \text{) },\\ &{}&{}&{}\\ &{} \ldots &{} &{}\\ &{}&{}&{}\\ \left( w_1, w_2\right) &{}=&{} \left( \frac{1}{3} , \frac{3}{5} \right) ,&{}\\ &{}&{}&{}\\ \left( w_1, w_2\right) &{}=&{} \left( \frac{1}{3} , \frac{7}{9} \right) ,&{}\\ &{}&{}&{}\\ \left( w_1, w_2\right) &{}=&{} \left( \frac{1}{3} , \frac{5}{3} \right) ,&{}\\ &{}&{}&{}\\ \left( w_1, w_2\right) &{}=&{} \left( \frac{1}{3} , 1 \right) &{} \text{(radiation } \text{ plus } \text{ stiff } \text{ fluid) },\\ &{}&{}&{}\\ \left( w_1, w_2\right) &{}=&{} \left( \frac{1}{3} , -\frac{1}{9} \right) ,&{}\\ &{}&{}&{}\\ \left( w_1, w_2\right) &{}=&{} \left( \frac{1}{3} , \frac{1}{15} \right) ,&{}\\ &{}&{}&{}\\ \left( w_1, w_2\right) &{}=&{} \left( \frac{1}{3} , \frac{1}{7} \right) ,&{}\\ &{}&{}&{}\\ &{} \ldots &{} &{}\\ &{}&{}&{}\\ \left( w_1, w_2\right) &{}=&{} \left( \frac{1}{3} , \frac{1}{3} \right) &{}\text{(again, } \text{ a } \text{ single } \text{ radiation } \text{ fluid) }. \end{array} \end{aligned}$$

#### $\Lambda$ plus a second (real or effective) fluid

Setting $w_2=-1$ corresponds to $m=0$ and Eq. (32) is satisfied for any $w_1\ne -1$.

#### Stiff matter plus a second (real or effective) fluid

Setting $w_1=1$ (the equation of state parameter of a stiff fluid) yields

45 $$\begin{aligned} w_2=\frac{2m-1}{2m+1} \end{aligned}$$

and the pairs

46 $$\begin{aligned} \begin{array}{rcll} \left( w_1, w_2 \right) &{}=&{} \left( 1, 1 \right) &{} \text{(single } \text{ stiff } \text{ fluid) },\\ &{}&{}&{}\\ &{} \ldots &{} &{}\\ &{}&{}&{}\\ \left( w_1, w_2 \right) &{}=&{} \left( 1, \frac{7}{5} \right) ,&{} \\ &{}&{}&{}\\ \left( w_1, w_2 \right) &{}=&{} \left( 1, \frac{5}{3} \right) ,&{}\\ &{}&{}&{}\\ \left( w_1, w_2 \right) &{}=&{} \left( 1, 3 \right) ,&{}\\ &{}&{}&{}\\ \left( w_1, w_2 \right) &{}=&{} \left( 1, -1 \right) &{} \text{(stiff } \text{ fluid } \text{ plus } \Lambda \text{) }, \\ &{}&{}&{}\\ \left( w_1, w_2 \right) &{}=&{} \left( 1, \frac{1}{3} \right) &{} \text{(stiff } \text{ fluid } \text{ plus } \text{ radiation) },\\ &{}&{}&{}\\ \left( w_1, w_2 \right) &{}=&{} \left( 1, \frac{3}{5} \right) ,&{}\\ &{}&{}&{}\\ \left( n, w_2 \right) &{}=&{} \left( 1, \frac{5}{7} \right) ,&{}\\ &{}&{}&{}\\ &{} \ldots &{} &{}\\ &{}&{}&{}\\ \left( w_1, w_2 \right) &{}=&{} \left( 1 , 1 \right) &{} \text{(again, } \text{ a } \text{ single } \text{ stiff } \text{ fluid) }. \end{array} \end{aligned}$$

## Luminosity distance

The luminosity distance versus redshift relation $$D_L(z)$$ is important to reconstruct the universe model from observations and has led to the discovery of the present acceleration of the cosmic expansion using type Ia supernovae [16–26]. In addition, the reciprocity relation $$D_L =\left( 1+z\right) ^2 D_A$$ between luminosity distance $$D_L $$ and area distance $$D_A$$ is used as a probe of fundamental cosmology [27].

The luminosity distance in a FLRW universe is expressed by an integral of the Chebyshev form (14) and can be calculated exactly in certain cases that we find below. First, let us review the derivation of $D_L(z)$ (e.g., [11, 28]).

Let us rewrite the FLRW line element as

47 $$\begin{aligned} ds^2 =- dt^2 +a^2(t) \left[ d\chi ^2 +S_k^2(\chi ) d\Omega _{(2)}^2 \right] , \end{aligned}$$

where $\chi$ is the hyperspherical radius and

48 $$\begin{aligned} S_k(\chi )= \left\{ \begin{array}{ccc} \sin \chi &{} \quad \text{ if } &{} K=1,\\ \chi &{} \quad \text{ if } &{} K=0,\\ \sinh \chi &{} \quad \text{ if } &{} K=-1.\end{array} \right. \end{aligned}$$

The luminosity distance $D_L$ between a light source and an observer is defined by

49 $$\begin{aligned} D_L^2 = \frac{L}{4\pi F}, \end{aligned}$$

where *L* is the absolute luminosity of the source and *F* is the flux density measured by the observer. Since

50 $$\begin{aligned} \frac{F}{L} =\frac{1}{A\left( 1+z\right) ^2} \end{aligned}$$

and the present area *A* of a sphere of hyperspherical radius $\chi$ is $A = 4\pi \, a_0^2 \, S_k^2(\chi )$, the luminosity distance becomes

51 $$\begin{aligned} D_L = \sqrt{ \frac{A(1+z)^2}{4\pi }} = \left( 1+z \right) \, a_0 \, S_k (\chi ). \end{aligned}$$

$\chi$ needs to be eliminated using

52 $$\begin{aligned} \chi = a_0^{-1} \int \frac{da}{a^2 \, H(a)} =a_0^{-1} \int _0^z \frac{dz'}{H(z')}, \end{aligned}$$

while the Einstein–Friedmann equation gives

53 $$\begin{aligned} H^2= & {} \frac{8\pi G}{3} \, \sum _i \rho _i -\frac{K}{a^2}\nonumber \\= & {} \frac{8\pi G}{3} \, \sum _i \rho _i - a_0^2 H_0^2 \left( \Omega _0^\mathrm {(tot)} -1\right) \end{aligned}$$

and $$\rho _i = \rho _{i0} \left( 1+z \right) ^{3(w_i+1)}$$. Then,

54 $$\begin{aligned} H^2 = H_0^2 \sum _i \, \frac{\rho _i}{\rho _c} + \left( 1-\Omega _0^\mathrm {(tot)} \right) a_0^2 \, H_0^2, \end{aligned}$$

where $\rho _c\equiv 3H^2 /(8\pi G)$ is the critical density. Finally,

55 $$\begin{aligned} H(z) = H_0 \sqrt{ \sum _i \Omega _{i0} \left( 1+z \right) ^{3(w_i+1)} +1-\Omega _0^\mathrm {(tot)} } \equiv H_0 \, E(z) \end{aligned}$$

gives $\chi = a_0^{-1} \int _0^z \frac{dz'}{E(z')}$ and the luminosity distance becomes

56 $$\begin{aligned} D_L(z) = (1+z) \, a_0 \, S_k \Bigg ( \frac{1}{a_0 H_0} \int _0^z \frac{dz'}{E(z')} \Bigg ). \end{aligned}$$

Since $a_0= H_0^{-1}/\sqrt{ |\Omega _{K0}|}$, where $\Omega _{K0}= -\frac{K}{a_0^2 H_0^2}$ is the curvature density at the present time in units of the critical density, we have

57 $$\begin{aligned} D_L (z) = \frac{(1+z) \, H_0^{-1} }{ \sqrt{ | \Omega _{K0}|} } \, S_K \Bigg ( \sqrt{ |\Omega _{K0}| } \, \int _0^z \frac{dz'}{E(z')} \Bigg ), \end{aligned}$$

which becomes particularly simple in a spatially flat universe

58 $$\begin{aligned} D_L^\mathrm {(flat)} (z) = (1+z) \, H_0^{-1} \, \int _0^z \frac{dz'}{E(z')}. \end{aligned}$$

Now the question is: can we express the integral

59 $$\begin{aligned} I \equiv \int _0^z \frac{dz'}{E(z')} = \int _0^z \frac{dz'}{\sqrt{ 1-\Omega _0^\mathrm {(tot)} + \sum _i \Omega _{i0} (1+z')^{3(w_i+1)} } } \end{aligned}$$

in terms of a finite number of elementary functions? This integral is similar to the one appearing in the lookback time (13), but now the limits of integration are 0 and *z* instead of 0 and 1.

### Single fluid

For a single fluid, using the variable $x \equiv (1+z)^{-1}$, we have

60 $$\begin{aligned} I_1&\equiv \int _0^z \frac{dz'}{\sqrt{1 - \Omega _0^\mathrm {(tot)} + \Omega _0(1+z')^{3(w+1)} }} \nonumber \\&= \int _x^1 dx' \left( x' \right) ^{-2} \left[ 1-\Omega _0^\mathrm {(tot)} +\Omega _0 (x')^{-3(w_1+1)} \right] ^{-1/2}, \end{aligned}$$

which is of the Chebyshev form (14) with $$ p=-2$$, $$r=-3(w+1)$$, $$ q=-1/2$$. Imposing that $$w\in {\mathbb {Q}}$$, it is

61 $$\begin{aligned} \frac{p+1}{r} = \frac{1}{3(w+1)}, \quad \frac{p+1}{r}+q = -\frac{(3w+1)}{6(w+1)} \end{aligned}$$

and $$ (p+1)/r = n \in {\mathbb {Z}}$$ implies

62 $$\begin{aligned}&w_n=\frac{1}{3n}-1 = -1, \ldots -\frac{10}{9}, -\frac{ 7}{6}, - \frac{4}{3} , -\frac{2}{3} , -\frac{5}{6}, \nonumber \\&\quad \qquad - \frac{8}{9}, \ldots , -1. \end{aligned}$$

The second condition $\frac{p+1}{r}+q=m\in {\mathbb {Z}}$ yields

63 $$\begin{aligned} w_m&= - \frac{(6m+1)}{3(2m+1)}= -1, \ldots , -\frac{1}{3}, -\frac{7}{9}, -\frac{5}{3}, \frac{13}{9}, \ldots , -1 \end{aligned}$$

or $$ n=0$$ and $$w=-1/3 $$, which corresponds to the Milne universe (Minkowski space with a hyperbolic foliation).

The situation of a single fluid plus cosmological constant $\Lambda$ is obtained with the replacement $1-\Omega _0^\mathrm {(tot)} \rightarrow 1- \Omega _0^\mathrm {(tot)} +\Omega _{\Lambda 0}$.

### Two fluids

Suppose that the FLRW universe is sourced by two fluids, then $D_L(z)$ depends on

64 $$\begin{aligned}&\int _0^z \frac{ dz'}{ \sqrt{ 1-\Omega _0^\mathrm {(tot)} +\Omega _0^{(1)} (1+z')^{3(w_1+1)} +\Omega _0^{(2)} (1+z')^{3(w_2+1)} } } \nonumber \\&\equiv I_2. \end{aligned}$$

For $K=0$ (or $\Omega _0^\mathrm {(tot)}=1$), corresponding to the luminosity distance (58), use the variable $y \equiv 1+z$ to obtain

65 $$\begin{aligned} I_2= & {} \int _1^y \frac{dy'}{\sqrt{ \Omega _0^{(1)} y^{ 3(w_1+1)} +\Omega _0^{(2)} \, y^{3(w_2+1)} }} \nonumber \\= & {} \int _1^y dy' \frac{ (y')^{\frac{-3(w_1+1)}{2} } }{\sqrt{ \Omega _0^{(1)} +\Omega _0^{(2)} (y')^{ 3(w_2-w_1)} } }, \end{aligned}$$

which is of the form (14) appearing in the Chebyshev theorem with $p=-3(w_1+1)/2$,  $q=-1/2$, and $r=3(w_2-w_1)$. Then

66 $$\begin{aligned} \frac{p+1}{r} = \frac{-(3w_1+1)}{6(w_2-w_1)}, \quad \quad \frac{p+1}{r} +q=\frac{-(3w_2+1)}{6(w_2-w_1)}. \end{aligned}$$

The first condition for “simple” integrability $(p+1)/r=n \in {\mathbb {Z}}$ gives

67 $$\begin{aligned} w_2= \frac{3(2n-1)w_1-1}{6n} \quad \text{ or } \quad w_1=-\frac{1}{3}, \quad \text{ any } \; w_2 \ne - \frac{1}{3}. \end{aligned}$$

If the first fluid is a dust ($w_1=0$), we obtain the pairs

68 $$\begin{aligned} \begin{array}{rcll} \left( w_1, w_2 \right) &{}=&{} \left( 0, 0 \right) &{} \text{(single } \text{ dust } \text{ fluid) },\\ &{}&{}&{}\\ &{} \ldots &{} &{}\\ &{}&{}&{}\\ \left( w_1, w_2 \right) &{}=&{} \left( 0, \pm \frac{1}{6} \right) ,&{} \\ &{}&{}&{}\\ \left( w_1, w_2 \right) &{}=&{} \left( 0, \pm \frac{1}{12} \right) ,&{}\\ &{}&{}&{}\\ \left( w_1, w_2 \right) &{}=&{} \left( 0, -\frac{1}{18} \right) ,&{}\\ &{}&{}&{}\\ \left( w_1, w_2 \right) &{}=&{} \left( 0, \frac{1}{9} \right) ,&{}\\ &{}&{}&{}\\ &{} \ldots &{} &{}\\ &{}&{}&{}\\ \left( w_1, w_2 \right) &{}=&{} \left( 0 , 0 \right) &{} \text{(again, } \text{ a } \text{ single } \text{ dust) }. \end{array} \end{aligned}$$

If instead the first fluid is radiation, $w_1=1/3$, we have the pairs

69 $$\begin{aligned} \left( w_1, w_2 \right)&= \left( \frac{1}{3}, \frac{1}{3} \right)&\text{(single } \text{ radiation } \text{ fluid) },\nonumber \\&\nonumber \\&\ldots&\nonumber \\&\nonumber \\ \left( w_1, w_2 \right)&= \left( \frac{1}{3}, 0 \right)&\text{(radiation } \text{ plus } \text{ dust) }, \nonumber \\&\nonumber \\ \left( w_1, w_2 \right)&= \left( \frac{1}{3}, \frac{2}{3} \right) ,&\nonumber \\&\nonumber \\ \left( w_1, w_2 \right)&= \left( \frac{1}{3}, \frac{1}{6} \right) ,&\nonumber \\&\nonumber \\ \left( w_1, w_2 \right)&= \left( \frac{1}{3}, \frac{1}{2} \right) ,&\nonumber \\&\nonumber \\ \left( w_1, w_2 \right)&= \left( \frac{1}{3}, \frac{2}{9} \right) ,&\nonumber \\&\nonumber \\ \left( w_1, w_2 \right)&= \left( \frac{1}{3}, \frac{4}{9} \right) ,&\nonumber \\&\nonumber \\&\ldots&\nonumber \\&\nonumber \\ \left( w_1, w_2 \right)&= \left( \frac{1}{3} , \frac{1}{3} \right)&\text{(again, } \text{ a } \text{ single } \text{ radiation } \text{ fluid) }. \end{aligned}$$

If the first fluid is stiff, $w_1=1$, the pairs giving Chebyshev integrability are

70 $$\begin{aligned} \begin{array}{rcll} \left( w_1, w_2 \right) &{}=&{} \left( 1, 1 \right) &{} \text{(single } \text{ stiff } \text{ fluid) },\\ &{}&{}&{}\\ &{} \ldots &{} &{}\\ &{}&{}&{}\\ \left( w_1, w_2 \right) &{}=&{} \left( 1, \frac{1}{3} \right) &{} \text{(stiff } \text{ fluid } \text{ plus } \text{ radiation) }, \\ &{}&{}&{}\\ \left( w_1, w_2 \right) &{}=&{} \left( 1, \frac{5}{3} \right) ,&{}\\ &{}&{}&{}\\ \left( w_1, w_2 \right) &{}=&{} \left( 1, \frac{2}{3} \right) ,&{}\\ &{}&{}&{}\\ \left( w_1, w_2 \right) &{}=&{} \left( 1, \frac{4}{3} \right) , &{} \\ &{}&{}&{}\\ \left( w_1, w_2 \right) &{}=&{} \left( 1, \frac{7}{9} \right) , &{}\\ &{}&{}&{}\\ &{} \ldots &{} &{}\\ &{}&{}&{}\\ \left( w_1, w_2 \right) &{}=&{} \left( 1 , 1 \right) &{} \text{(again, } \text{ a } \text{ single } \text{ stiff } \text{ fluid) }. \end{array} \end{aligned}$$

If the first (effective) fluid is the cosmological constant, $w_1=-1$, we have instead the pairs

71 $$\begin{aligned} \left( w_1, w_2 \right)&= \left( -1, 1 \right)&( \Lambda \; \text{ and } \text{ no } \text{ matter}),\nonumber \\&\nonumber \\&\ldots&\nonumber \\&\nonumber \\ \left( w_1, w_2 \right)&= \left( -1, -\frac{2}{3} \right)&\text{(stiff } \text{ fluid } \text{ plus } \text{ radiation) },\nonumber \\&\nonumber \\ \left( w_1, w_2 \right)&= \left( -1, -\frac{4}{3} \right) ,&\nonumber \\&\nonumber \\ \left( w_1, w_2 \right)&= \left( -1, -\frac{5}{6} \right) ,&\nonumber \\&\nonumber \\ \left( w_1, w_2 \right)&= \left( -1, -\frac{7}{6} \right) ,&\nonumber \\&\nonumber \\&\ldots&\nonumber \\&\nonumber \\ \left( w_1, w_2 \right)&= \left( -1 , -1 \right)&\text{(again }\, \Lambda \, \text{ and } \text{ no } \text{ matter}). \end{aligned}$$

The second condition for integrability à la Chebyshev

72 $$\begin{aligned} \frac{p+1}{r} +q=\frac{-(3w_2+1)}{6(w_2-w_1)} =m \in {\mathbb {Z}} \end{aligned}$$

gives

73 $$\begin{aligned} w_2=\frac{6mw_1-1}{3(2m+1)} \quad \text{ or }\quad w_2=-\frac{1}{3}, \quad \text{ any }\, w_1\ne -\frac{1}{3}. \end{aligned}$$

In particular, if the first fluid is a dust, $w_1=0$, we have $w_2=-\left( [ 3(2m+1)\right] ^{-1}$ and the pairs

74 $$\begin{aligned} \begin{array}{rcll} \left( w_1, w_2 \right) &{}=&{} \left( 0, 0 \right) &{} \text{(single } \text{ dust } \text{ fluid) },\\ &{}&{}&{}\\ &{} \ldots &{} &{}\\ &{}&{}&{}\\ \left( w_1, w_2 \right) &{}=&{} \left( 0, \pm \frac{1}{3} \right) ,&{} \\ &{}&{}&{}\\ \left( w_1, w_2 \right) &{}=&{} \left( 0, \pm \frac{1}{9} \right) ,&{}\\ &{}&{}&{}\\ \left( w_1, w_2 \right) &{}=&{} \left( 0, \pm \frac{1}{15} \right) ,&{}\\ &{}&{}&{}\\ &{} \ldots &{} &{}\\ &{}&{}&{}\\ \left( w_1, w_2 \right) &{}=&{} \left( 0 , 0 \right) &{} \text{(again, } \text{ a } \text{ single } \text{ dust) }. \end{array} \end{aligned}$$

If the first fluid is radiation, $w_1=1/3$, then $w_2=\frac{2m-1}{3(2m+1)}$, giving the pairs

75 $$\begin{aligned} \left( w_1, w_2 \right)&= \left( \frac{1}{3}, \frac{1}{3} \right)&\text{(single } \text{ radiation } \text{ fluid) },\nonumber \\&\nonumber \\&\ldots&\nonumber \\&\nonumber \\ \left( w_1, w_2 \right)&= \left( \frac{1}{3}, \pm 1 \right)&(\text{ radiation } \text{ plus } \text{ dust } \text{ or }\, \Lambda ) , \nonumber \\&\nonumber \\ \left( w_1, w_2 \right)&= \left( \frac{1}{3}, -\frac{1}{3} \right) ,&\nonumber \\&\nonumber \\ \left( w_1, w_2 \right)&= \left( \frac{1}{3}, \frac{1}{9} \right) ,&\nonumber \\&\nonumber \\ \left( w_1, w_2 \right)&= \left( \frac{1}{3}, \frac{1}{5} \right) ,&\nonumber \\&\nonumber \\ \left( w_1, w_2 \right)&= \left( \frac{1}{3}, \frac{5}{9} \right) ,&\nonumber \\&\nonumber \\ \left( w_1, w_2 \right)&= \left( \frac{1}{3}, \frac{5}{21} \right) ,&\nonumber \\&\nonumber \\&\ldots&\nonumber \\&\nonumber \\ \left( w_1, w_2 \right)&= \left( \frac{1}{3} , \frac{1}{3} \right)&\text{(again, } \text{ a } \text{ single } \text{ radiation } \text{ fluid) }. \end{aligned}$$

If the first fluid is stiff with $w_1=1$, then $w_2= \frac{6m-1}{3(2m+1)}$, generating the pairs

76 $$\begin{aligned} \begin{array}{rcll} \left( w_1, w_2 \right) &{}=&{} \left( 1, 1 \right) &{} \text{(single } \text{ stiff } \text{ fluid) },\\ &{}&{}&{}\\ &{} \ldots &{} &{}\\ &{}&{}&{}\\ \left( w_1, w_2 \right) &{}=&{} \left( 1, -\frac{1}{3} \right) &{} \text{(stiff } \text{ fluid } \text{ plus } \text{ radiation) }, \\ &{}&{}&{}\\ \left( w_1, w_2 \right) &{}=&{} \left( 1, \frac{1}{9} \right) ,&{}\\ &{}&{}&{}\\ \left( w_1, w_2 \right) &{}=&{} \left( 1, \frac{7}{13} \right) ,&{}\\ &{}&{}&{}\\ \left( w_1, w_2 \right) &{}=&{} \left( 1, \frac{11}{15} \right) , &{} \\ &{}&{}&{}\\ \left( w_1, w_2 \right) &{}=&{} \left( 1, \frac{13}{9} \right) , &{}\\ &{}&{}&{}\\ &{} \ldots &{} &{}\\ &{}&{}&{}\\ \left( w_1, w_2 \right) &{}=&{} \left( 1 , 1 \right) &{} \text{(again, } \text{ a } \text{ single } \text{ stiff } \text{ fluid) }. \end{array} \end{aligned}$$

As an example, consider the case of a spatially flat FLRW universe filled with radiation and dust, $w_1=1/3$ and $w_2=0$ appearing in the list (37), in which case

77 $$\begin{aligned} I_2= & {} \int _1^y dy' \, \frac{ (y')^{-2}}{ \sqrt{ \Omega _0^{(1)} +\Omega _0^{(2)} /y'}}\nonumber \\= & {} -\frac{2}{\Omega _0^{(2)} } \sqrt{ \Omega _0^{(1)} +\frac{\Omega _0^{(2)}}{y'} } \, \Bigg |_1^y \nonumber \\= & {} \frac{2}{\Omega _0^{(2)} } \left[ \sqrt{ \Omega _0^{(1)} +\Omega _0^{(2)} } - \sqrt{ \Omega _0^{(1)} + \frac{\Omega _0^{(2)}}{y} } \, \right] \end{aligned}$$

and the luminosity distance versus redshift relation is

78 $$\begin{aligned} D_L(z)= & {} \frac{2 H_0^{-1} \left( 1+z\right) }{\Omega _0^\mathrm {(dust)} }\nonumber \\&\times \left[ \sqrt{ \Omega _0^\mathrm {(rad)} +\Omega _0^\mathrm {(dust)} }- \sqrt{ \Omega _0^\mathrm {(rad)} + \frac{\Omega _0^\mathrm {(dust)}}{1+z} } \,\, \right] .\nonumber \\ \end{aligned}$$

It is unfortunate that the $\Lambda$CDM model corresponding to $\Lambda$ and dust is not integrable à la Chebyshev. Usually, the luminosity distance $D_L(z)$ is expanded for small *z* to compare it with type Ia supernovae data, However, standard candles at redshifts $z\sim 1$ are present in current catalogues and the small *z* expansion fails for those objects, hence the search for new parametrizations valid at high redshifts [29–31].

## Conclusions

It is of interest to know when the lookback time $t_L$, the age $t_0$ of the universe, and the luminosity distance versus redshift $D_L(z)$ can be computed analytically in FLRW cosmology. These quantities contain integrals expressed by hypergeometric series, which truncate to a finite number of terms under certain conditions expressed by the Chebyshev theorem of integration [2, 3]. We have classified the situations in which the Chebyshev theorem holds for a FLRW universe containing real or effective fluids (including curvature and the cosmological constant). The Chebyshev theorem is not useful for situations with more than three fluids or effective fluids. Moreover, when the universe is dominated by a single fluid for most of its history, one can approximate the age of the universe with the duration of the epoch dominated by that fluid (for example, in a universe containing only dust and radiation, with $\Lambda =0$, neglecting the duration of the radiation-dominated age only introduces a small error in the age computed using only dust).

For example, a model of the universe called “$$R_h=ct $$ universe” was proposed recently by Melia and collaborators [35–40]. This model invokes an exotic fluid with equation of state parameter $$w=-1/3$$ that is *not* spatial curvature^3^ to achieve an (at least approximately) coasting universe [35–40]. This proposal is still rather preliminary and a realistic model will have to include galaxies and dark matter, usually modelled as dust. This more refined model will contain two fluids with equation of state parameters $$w_1=0$$ and $$w_2=-1/3$$ and this is precisely one of the integrability cases in which the age of the universe and luminosity distance can be computed exactly in simple form (cf. Eqs. (42), (73), and (74)). To wit, the age of the universe $$t_0$$ in this case is given by the simple expression

79 $$\begin{aligned} t_0 H_0= & {} \int _0^1 dx \, \sqrt{ \frac{x}{ \Omega _0^{(1)} + \Omega _0^{(2)} \, x} } \nonumber \\= & {} \sqrt{ \frac{\Omega _0^{(1)} }{ \left( \Omega _0^{(2)}\right) ^2 } +1 } - \frac{ \Omega _0^{(1)} }{ \left( \Omega _0^{(2)} \right) ^{3/2} } \, \sinh ^{-1} \sqrt{ \frac{ \Omega _0^{(2)} }{\Omega _0^{(1)} } }. \end{aligned}$$

The corresponding integral relevant to calculate the luminosity distance $D_L(z)$ is again given by a simple expression,

80 $$\begin{aligned} I_2= & {} \int _1^y \frac{ds}{s \sqrt{ \Omega _0^{(1)} s + \Omega _0^{(2)} }} \nonumber \\= & {} -2 \left[ \tanh ^{-1} \left( \sqrt{ \frac{ \Omega _0^{(2)} }{ \Omega _0^{(1)} \,s + \Omega _0^{(2)} }} \right) \, \right] _1^y \end{aligned}$$

where $y=z+1$. As a result, the luminosity distance (58) is

81 $$\begin{aligned} D_L(z)= & {} 2H_0^{-1} \left( 1+z \right) \nonumber \\&\times \,\left( \tanh ^{-1} \sqrt{ \Omega _0^{(2)} } -\tanh ^{-1} \sqrt{ \frac{\Omega _0^{(2)} }{ 1 + \Omega _0^{(1)} z } } \,\, \right) .\nonumber \\ \end{aligned}$$

As another example, a coasting period of the universe was considered, e.g., in [41] to help structure formation and it was of interest to compute the age of the universe up to that stage, and the loitering time, i.e., the period of time that the universe spends in the loitering stage. Scenarios were obtained by including in the universe, in addition to dust, a second fluid with negative equation of state $$w_2=-m/3$$, with *m* integer [41]. To this regard, it is well-known that a network of non-intercommuting topological defects produces an effective equation of state parameter $$w = - m/3$$, where *m* is the dimension of the defect [42, 43]. In particular, domain walls yield $$ w = -2/3 $$ while a frustrated cosmic string network gives $$w = -1/3 $$ [44, 45]. Although no longer competitive with the $$\Lambda $$CDM model in many regards, such theoretical models resurface from time to time in theoretical studies to test new ideas before attempting to implement them in a realistic cosmological model. *Vice-versa*, if one has freedom to choose a range of cosmological models to test a theoretical idea, one now knows which models will give simple analytical answers for $$t_0$$ and $$D_L(z)$$.

In cosmography, the luminosity distance versus redshift relation has been instrumental in detecting the acceleration of the cosmic expansion with type Ia supernovae [16–26] and is one of the most important observational relations. Building observational plots of $$D_L$$ versus *z* relies on expanding the relation $$D_L(z)$$ to second order around the present time and measuring the present values $$H_0 \equiv {\dot{a}}/a \Big |_0$$ of the Hubble function and $$q_0 \equiv - \ddot{a} a / {\dot{a}}^2 \Big |_0 $$ of the deceleration parameter (the third and fourth order terms in the series or, equivalently, the jerk and the snap are subject to much larger uncertainties). When distant objects at redshift $$z \sim 1 $$ are included in the samples, the expansion breaks down and one has to resort to alternative parametrizations, for example the Chevallier–Polarski–Linder (CPL) [29, 30] or the Cattoen–Visser [31] parametrizations. The Cattoen–Visser parametrization uses the parameter $$y\equiv z/(z+1) = 1-a/a_0 $$ and the fact that this is smaller than the redshift *z* causes the errors in the fitted parameters in the expansion to be larger [32] and has serious implications for the Hubble, and other, tensions afflicting the $$\Lambda $$CDM model [1]. While the CPL model seems superior for fitting the cosmic microwave background in comparison with other $$ \left( w_0, w_a \right) $$ models, it is not at low redshifts [33]. At higher redshifts ($$z \ge 2$$), it is still difficult to recover the $$\Lambda $$CDM model: a fifth order polynomial in *y* leads to 15% discrepancies in the luminosity distance (see Fig. 9 of Ref. [34]), or to 5% errors at $$ z = 1$$, hence cosmography beyond redshift 1 must include a large number of terms in the expansion.

Being able to compute $D_L(z)$ exactly is complementary to the cosmographic and numerical approaches. Unfortunately, among the infinitely many cases in which integration à la Chebyshev is possible, only a few correspond to physically realistic situations or even (real or effective) realistic fluids. Nevertheless, one wants to know when simple analytical expressions of $t_0$ and $D_L(z)$ exist. Even when they do not describe realistic epochs of the history of the universe, these situations can be used as toy models for theoretical purposes or for testing parametrizations in cosmography or numerical evaluations of $t_L$, $t_0$, and $D_L(z)$.

## Data Availability

This manuscript has no associated data or the data will not be deposited. [Authors’ comment: ...].

## Appendix A: Lookback time and age for $K=0$, $\Lambda \ne 0$, and a single fluid

The lookback time (15) integrates to

A.1 $$\begin{aligned} t_L= & {} \frac{2H_0^{-1} }{ 3(w+1)\sqrt{\Omega _{\Lambda 0}} } \coth ^{-1} \left( \frac{ \sqrt{ \Omega _{\Lambda 0} +\Omega _0 \, x^{-3(w+1)} } }{ \sqrt{\Omega _{\Lambda 0} }} \right) \Bigg |_{x_e}^1.\nonumber \\ \end{aligned}$$

Using the identity

A.2 $$\begin{aligned} \coth ^{-1} z=\frac{1}{2} \, \ln \left( \frac{z+1}{z-1} \right) \end{aligned}$$

for $|z|>1$, we have

A.3

In the limit $x_e\rightarrow 0$ one finds

A.4 $$\begin{aligned} t_L\rightarrow & {} t_0 = \frac{H_0^{-1} }{3(w+1)\sqrt{\Omega _{\Lambda 0} } } \, \ln \left( \frac{ 1+ \sqrt{ \Omega _{\Lambda 0}} }{ 1-\sqrt{ \Omega _{\Lambda 0} }} \right) \nonumber \\= & {} \frac{2H_0^{-1}}{3(w+1)\sqrt{\Omega _{\Lambda 0}}} \, \frac{1}{2} \, \ln \left[ \frac{ \left( 1+ \sqrt{ \Omega _{\Lambda 0}} \right) ^2 }{ 1- \Omega _{\Lambda 0} } \right] \nonumber \\= & {} \frac{2H_0^{-1}}{3(w+1)\sqrt{\Omega _{\Lambda 0}}} \, \ln \left( \frac{ 1+ \sqrt{ \Omega _{\Lambda 0}} }{ \sqrt{ 1- \Omega _{\Lambda 0} }} \right) . \end{aligned}$$

## Footnotes

1 If $\Lambda =0$ the integration is trivial.

2 This relation can also be derived from the geodesic deviation equation for null geodesics [14].

3 That is, the three-dimensional space has the topology of ${\mathbb {R}}^3$.

## References

[1] L. Verde, T. Treu, A.G. Riess, Tensions between the early and the late universe. Nat. Astron. **3**, 891. 10.1038/s41550-019-0902-0. arXiv:1907.10625 [astro-ph.CO]

[2] P.L. Chebyshev, Sur l’integration des différentielles irrationnelles. J. Math. (Ser. 1) **18**, 87–111 (1853)

[3] Marchisotto EA, Zakeri G-A (1994). An invitation to integration in finite terms. Coll. Math. J..

[4] Jacobs KC (1968). Spatially homogeneous and Euclidean cosmological models with shear. Astrophys. J..

[5] Vajk JP (1969). Exact Robertson Walker cosmological solutions containing relativistic fluids. J. Math. Phys..

[6] McIntosh CBG (1972). I. Robertson–Walker metric. Aust. J. Phys..

[7] McIntosh CBG, Foyster JM (1972). Cosmological models with two fluids II. Conformal and conformally flat metrics. Aust. J. Phys..

[8] S. Chen, G. W. Gibbons, Y. Li, Y. Yang, Friedmann’s equations in all dimensions and Chebyshev’s theorem. JCAP **12**, 035 (2014). 10.1088/1475-7516/2014/12/035. arXiv:1409.3352 [astro-ph.CO]

[9] V. Faraoni, S. Jose, S. Dussault, Multi-fluid cosmology in Einstein gravity: analytical solutions. Gen. Relativ. Gravit. **53**(12), 109 (2021). 10.1007/s10714-021-02879-z. arXiv:2107.12488 [gr-qc]

[10] Wald RM (1984). General Relativity.

[11] Ellis GFR, Maartens R, MacCallum MAH (2012). Relativistic Cosmology.

[12] Kolb EW, Turner MS (1990). The Early Universe.

[13] V. Mukhanov, *Physical Foundations of Cosmology* (Cambridge University Press, Cambridge, 2005). 10.1017/CBO9780511790553

[14] G.F.R. Ellis, H. van Elst, Cosmological models: Cargese lectures 1998. NATO Sci. Ser. C **541**, 1–116 (1999). 10.1007/978-94-011-4455-1_1. arXiv:gr-qc/9812046

[15] Mattig W (1958). Über der zusammenhang zwischen rotverschiebung und scheinrare helligkeit. Astron. Nachr..

[16] A.G. Riess et al. [Supernova Search Team], Observational evidence from supernovae for an accelerating universe and a cosmological constant,. Astron. J. **116**, 1009–1038 (1998). 10.1086/300499. arXiv:astro-ph/9805201

[17] S. Perlmutter et al. [Supernova Cosmology Project], Measurements of $$\Omega $$ and $$\Lambda $$ from 42 high redshift supernovae. Astrophys. J. **517**, 565–586 (1999). 10.1086/307221. arXiv:astro-ph/9812133

[18] A.V. Filippenko, A.G. Riess, Results from the high Z supernova search team. Phys. Rep. **307**, 31–44 (1998). 10.1016/S0370-1573(98)00052-0. arXiv:astro-ph/9807008

[19] A.G. Riess, A.V. Filippenko, W. Li, B.P. Schmidt, An indication of evolution of type Ia supernovae from their risetimes. Astron. J. **118**, 2668–2674 (1999). 10.1086/301144. arXiv:astro-ph/9907038

[20] A.G. Riess, The case for an accelerating universe from supernovae. Publ. Astron. Soc. Pac. **112**, 1284 (2000). 10.1086/316624. arXiv:astro-ph/0005229

[21] A.G. Riess et al. [Supernova Search Team], The farthest known supernova: support for an accelerating universe and a glimpse of the epoch of deceleration. Astrophys. J. **560**, 49–71 (2001). 10.1086/322348. arXiv:astro-ph/0104455

[22] J.L. Tonry et al. [Supernova Search Team], Cosmological results from high-z supernovae. Astrophys. J. **594**, 1–24 (2003). 10.1086/376865. arXiv:astro-ph/0305008

[23] R.A. Knop et al. [Supernova Cosmology Project], New constraints on $$\Omega _M, \Omega _{\Lambda }$$, and $$w$$ from an independent set of eleven high-redshift supernovae observed with HST. Astrophys. J. **598**, 102 (2003). 10.1086/378560. arXiv:astro-ph/0309368

[24] B.J. Barris, J.L. Tonry, S. Blondin, P. Challis, R. Chornock, A. Clocchiatti, A.V. Filippenko, P. Garnavich, S.T. Holland, S. Jha et al., 23 High redshift supernovae from the IFA Deep Survey: doubling the SN sample at z $$>$$ 0.7. Astrophys. J. **602**, 571–594 (2004). 10.1086/381122. arXiv:astro-ph/0310843

[25] A.G. Riess et al. [Supernova Search Team], Type Ia supernova discoveries at z $$>$$ 1 from the Hubble Space Telescope: Evidence for past deceleration and constraints on dark energy evolution. Astrophys. J. **607**, 665–687 (2004). 10.1086/383612. arXiv:astro-ph/0402512

[26] A.G. Riess, The expansion of the universe is faster than expected. Nat. Rev. Phys. **2**(1), 10–12 (2019). 10.1038/s42254-019-0137-0. arXiv:2001.03624 [astro-ph.CO]

[27] B.A. Bassett, M. Kunz, Cosmic distance-duality as a probe of exotic physics and acceleration. Phys. Rev. D **69**, 101305 (2004). 10.1103/PhysRevD.69.101305. arXiv:astro-ph/0312443

[28] Carroll S (2004). An Introduction to General Relativity.

[29] M. Chevallier, D. Polarski, Accelerating universes with scaling dark matter. Int. J. Mod. Phys. D **10**, 213–224 (2001). 10.1142/S0218271801000822. arXiv:gr-qc/0009008

[30] E.V. Linder, Exploring the expansion history of the universe. Phys. Rev. Lett. **90**, 091301 (2003). 10.1103/PhysRevLett.90.091301. arXiv:astro-ph/0208512 10.1103/PhysRevLett.90.091301 12689209

[31] C. Cattoen, M. Visser, The Hubble series: convergence properties and redshift variables. Class. Quantum Gravity **24**, 5985–5998 (2007). 10.1088/0264-9381/24/23/018. arXiv:0710.1887 [gr-qc]

[32] V.C. Busti, Á. de la Cruz-Dombriz, P.K.S. Dunsby, D. Sáez-Gómez, Is cosmography a useful tool for testing cosmology? Phys. Rev. D **92**(12), 123512 (2015). 10.1103/PhysRevD.92.123512. arXiv:1505.05503 [astro-ph.CO]

[33] E.Ó. Colgáin, M.M. Sheikh-Jabbari, L. Yin, Can dark energy be dynamical?. Phys. Rev. D **104**(2), 023510 (2021). 10.1103/PhysRevD.104.023510. arXiv:2104.01930 [astro-ph.CO]

[34] T. Yang, A. Banerjee, E.Ó. Colgáin, Cosmography and flat $$\Lambda $$CDM tensions at high redshift. Phys. Rev. D **102**(12), 123532 (2020). 10.1103/PhysRevD.102.123532. arXiv:1911.01681 [astro-ph.CO]

[35] Melia F, Abdelqader M (2009). The cosmological spacetime. Int. J. Mod. Phys. D.

[36] F. Melia, A. Shevchuk, The $$R_h = ct$$ Universe. Mon. Not. R. Astron. Soc. **419**, 2579–2586 (2012). 10.1111/j.1365-2966.2011.19906.x. arXiv:1109.5189 [astro-ph.CO]

[37] F. Melia, The $$R_h=ct$$ universe without inflation. Astron. Astrophys. **553**, A76 (2013). 10.1051/0004-6361/201220447. arXiv:1206.6527 [astro-ph.CO]

[38] F. Melia, On recent claims concerning the $$R_h=ct$$ universe. Mon. Not. R. Astron. Soc. **446**, 1191–1194 (2015). 10.1093/mnras/stu2181. arXiv:1406.4918 [astro-ph.CO]

[39] F. Melia, Definitive test of the $$R_h = ct$$ universe using redshift drift. Mon. Not. R. Astron. Soc. **463**(1), L61–L63 (2016). 10.1093/mnrasl/slw157. arXiv:1608.00047 [astro-ph.CO]

[40] F. Melia, A comparison of the $$R_h = ct$$ and $$\Lambda $$CDM cosmologies using the cosmic distance duality relation. Mon. Not. R. Astron. Soc. **481**(4), 4855–4862 (2018). 10.1093/mnras/sty2596. arXiv:1804.09906 [astro-ph.CO]

[41] Sahni V, Feldman H, Stebbins A (1992). Loitering universe. Astrophys. J..

[42] Vilenkin A (1984). String dominated universe. Phys. Rev. Lett..

[43] D. Spergel, U.L. Pen, Cosmology in a string dominated universe. Astrophys. J. Lett. **491**, L67–L71 (1997). 10.1086/311074. arXiv:astro-ph/9611198

[44] R.A. Battye, M. Bucher, D. Spergel, Domain wall dominated universes. arXiv:astro-ph/9908047

[45] M. Bucher, D.N. Spergel, Is the dark matter a solid? Phys. Rev. D **60**, 043505 (1999). 10.1103/PhysRevD.60.043505. arXiv:astro-ph/9812022

